# Spontaneous Regression of Primary Melanoma and Multiple Melanocytic Nevi in a Patient With Metastatic Melanoma

**DOI:** 10.5826/dpc.1003a52

**Published:** 2020-06-29

**Authors:** Giovanni Paolino, Nathalie Rizzo, Riccardo Pampena, Pietro Bearzi, Alessandra Bulotta, Vanesa Gregorc, Pina Brianti, Elisa Moliterni, Santo Raffaele Mercuri

**Affiliations:** 1 Unit of Dermatology, IRCCS Ospedale San Raffaele, Milan, Italy; 2 Dermatologic Clinic, La Sapienza University of Rome, Italy; 3 Department of Pathology, IRCCS Ospedale San Raffaele, Milan, Italy; 4 Centro Oncologico ad Alta Tecnologia Diagnostica, Azienda Unità Sanitaria Locale—IRCCS di Reggio Emilia, Italy; 5 Department of Medical Oncology, IRCCS Ospedale San Raffaele, Milan, Italy

**Keywords:** cutaneous melanoma, regression, dermoscopy, dysplastic nevi, dermatopathology

## Introduction

Regression within cutaneous melanoma has been previously described as a spontaneous or therapy-induced phenomenon, driven by the activation of the immune system [1]. However, simultaneous development of regression in both melanoma and multiple melanocytic nevi is an extremely rare event, having been reported only twice in the literature [1,2].

## Case Presentation

**Figure 1:**
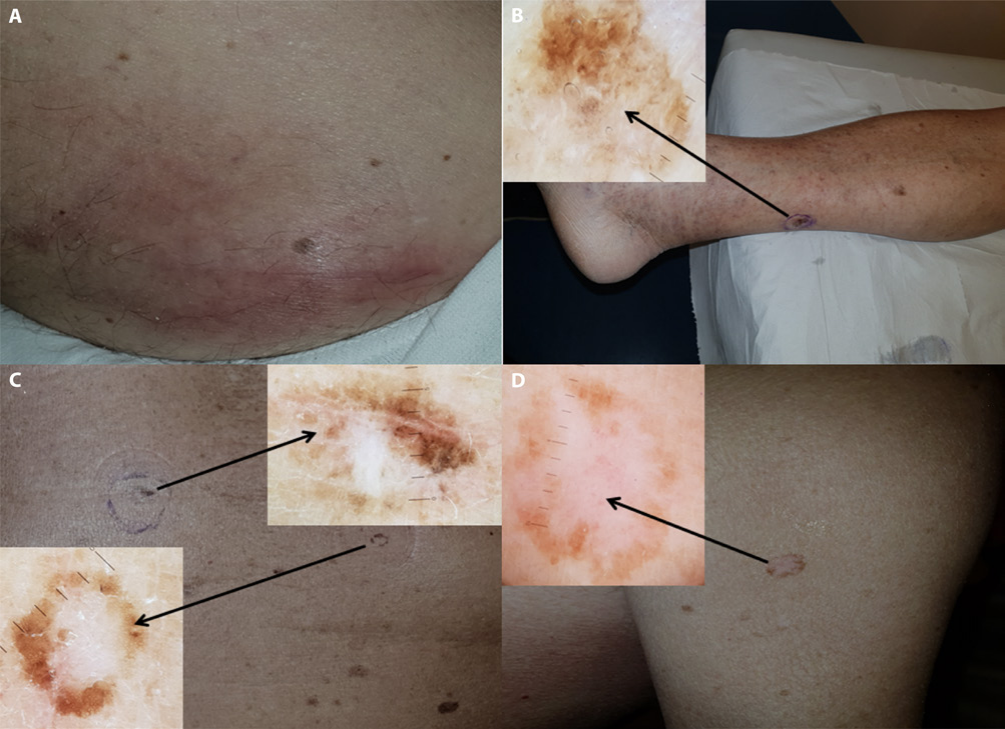
(A) Right inguinal lymphadenopathy with overlying inflammation. Histology revealed it was a nodal melanoma metastasis. (B) Pigmented lesion on the right calf (dimensions: 1.5 × 1 cm), with no sign of ulceration. Upper left insert: dermoscopy revealed asymmetric pigmentation, white central areas with scar-like depigmentation and mild peppering. Histopathological diagnosis: invasive malignant melanoma (see Figure 2, A and B). (C) Pigmented lesions on the right abdomen (diameter: 5 mm each) with clinical signs of regression. Upper right insert: dermoscopy showed central scar-like depigmentation and asymmetric globules on the right part of the lesion. Histological diagnosis: dysplastic nevus (see Figure 2C). Lower left insert: other pigmented lesion on the abdomen, showing similar clinical and dermoscopic features. Histological diagnosis: dysplastic nevus. (D) Pigmented lesion on the right upper arm (diameter: 1 cm) with signs of regression. Upper left insert: dermoscopy revealed central scar-like depigmentation, with minimal central ectatic vessels and a peripheral brown pigmentation. Histology: dysplastic nevus (see Figure 2D).

A 76-year-old Caucasian man came to our attention for lymphadenopathy of a 3-cm right inguinal lymph node (Figure 1A), subsequently diagnosed histopathologically as nodal melanoma metastasis.

Upon total body skin examination, multiple suspicious lesions on the trunk and limbs showed dermoscopic evidence of regression. Four of the lesions met additional atypical dermoscopic criteria and were excised to rule out primary melanoma (Figure 1, B–D).

**Figure 2:**
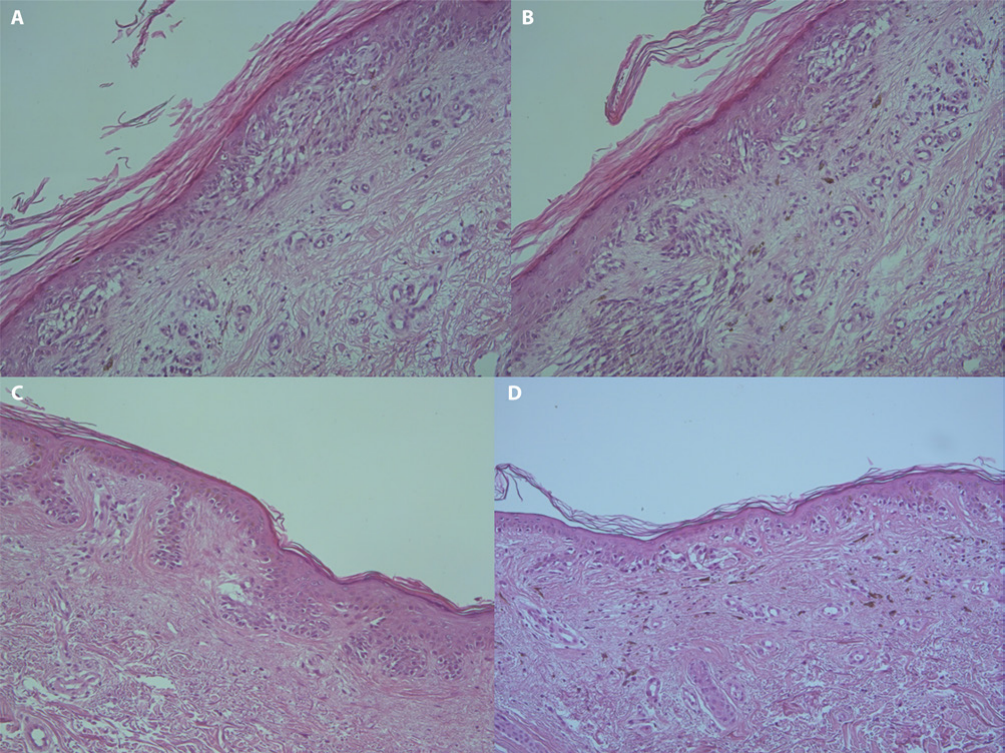
(A) The histology of the excised lesion on the right lower limb, revealed to be an invasive malignant melanoma. Breslow thickness: 0.3 mm (H&E, ×100). (B) Another histological specimen of the melanoma of panel A: regression >75%, signs of fibrosis in the superficial dermis, scattered melanophages, and patchy lymphocytic infiltration of the stroma (H&E, ×100). (C) The excised pigmented lesion on the right abdomen (upper right insert of Figure 1C) resulted in a dysplastic nevus with signs of regression and fibrosis in the upper dermis (H&E, ×100). (D) The excised pigmented lesion on the right upper arm (Figure 1D) resulted in a dysplastic nevus: signs of regression, fibrosis of the upper dermis, and scattered melanophages (H&E, ×100).

The excised lesions were flat and located on the right lower limb, right abdomen, and right upper arm. The histopathological diagnosis of the right lower limb lesion (ipsilateral to the nodal metastasis) was a primary melanoma with Breslow thickness of 0.3 mm, no ulceration, wide regression (>75%), and fibrosis of the superficial dermis (Figure 2, A and B). The remaining 3 lesions were dysplastic nevi with regression and fibrosis (Figure 2, C and D).

A total body CT scan showed brain, adrenal, lymphatic, and bone metastases. As the nodal metastasis proved BRAF (V600K, V600R, V600M) positive, combined target therapy with BRAF and MEK inhibitors ensued. At 6 months follow-up, metastases showed mild regression and no other melanocytic nevi developed regression features.

## Conclusions

Regression in melanoma occurs 6 times more often than in other malignancies and relates to melanocytes’ elevated immunogenicity [1]. Indeed, circulating antibodies against melanocyte cytoplasmic proteins have been isolated in melanoma patients and tumor-specific CD8+ T cells are present in melanoma-associated vitiligo [1]. Moreover, in vitro cytotoxic T lymphocytes from melanoma tissue have been shown to target differentiation antigens shared with normal melanocytes [1].

We suggest that the presence of multiple metastases in our case induced a vigorous immune response, leading to regression of the primary melanoma (right lower limb) and other melanocytic lesions sharing the same antigens. This is confirmed by the presence of dermoscopic and histological regression in the primary melanoma and the excised dysplastic nevi.

The role of regression in the prognosis of melanoma is debated [1]. Indeed, there is still no consensus as to whether the regression is associated with a worse or better prognosis. On one hand, the presence of wide regression (>75%) may “hide” the primary melanoma, eventually delaying the diagnosis with a consequent worse prognosis [2]. Furthermore, since melanoma cells can escape T-cell-mediated destruction by the production of immunosuppressive cytokines and the downregulation of HLA class I [1], metastasis can easily spread in melanomas with wide regression. On the other hand, T lymphocytes may proliferate until the antigenic trigger (melanoma cells) is present [2]. Finally, as reported in the literature [2], the presence of regression increases the likelihood of a positive BRAF mutation.

In conclusion, the simultaneous presence of regression in melanoma and benign melanocytic lesions results from the immune response against melanocytic antigens expressed by both cell lineages [1,2]. Dermoscopic regression in multiple melanocytic lesions should increase awareness for malignancy. Primary melanoma regression >75% in the primary melanoma increases the risk for metastasis, due to Breslow thickness underestimation and the capability of melanoma to escape tumor surveillance [1,2].
